# Supplementary material for: Room-temperature direct synthesis of semi-conductive PbS nanocrystal inks for optoelectronic applications
Source: Nat Commun. 2019 Nov 13;10:5136. doi: 10.1038/s41467-019-13158-6 (PMC6853884; doi:10.1038/s41467-019-13158-6)
Supplement: Supplementary file 1 — Supplementary Information [file 41467_2019_13158_MOESM1_ESM.pdf]

Supplementary Information

Room-temperature direct synthesis of semi-conductive PbS nanocrystal inks for optoelectronic applications

Wang et al.

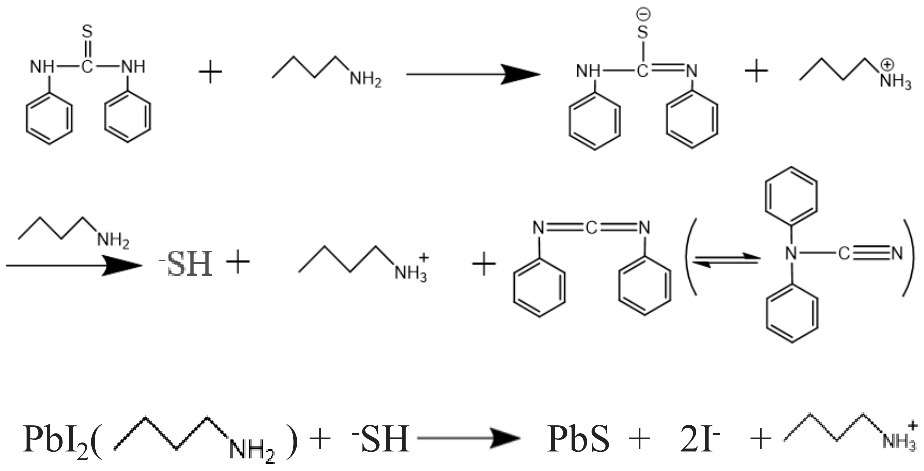

Supplementary Figure 1. Scheme diagram for the reaction between diphenyl thiourea (DPhTA), butylamine (BA) and lead iodide (PbI_2).

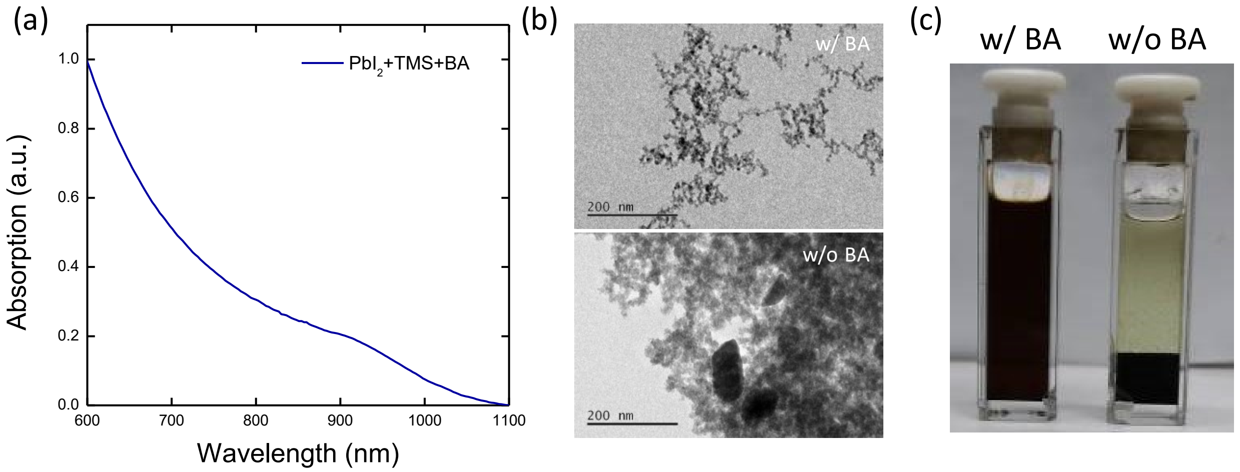

Supplementary Figure 2. PbS-I NCs direct-synthesized with hexamethyl disilathiane (TMS) as the sulfur precursor. (a) Absorption spectrum of PbS NCs synthesized with butylamine (BA). (b) TEM image of PbS NCs (w/ and w/o BA). (c) Photos of reaction solution with and without butyl amine.

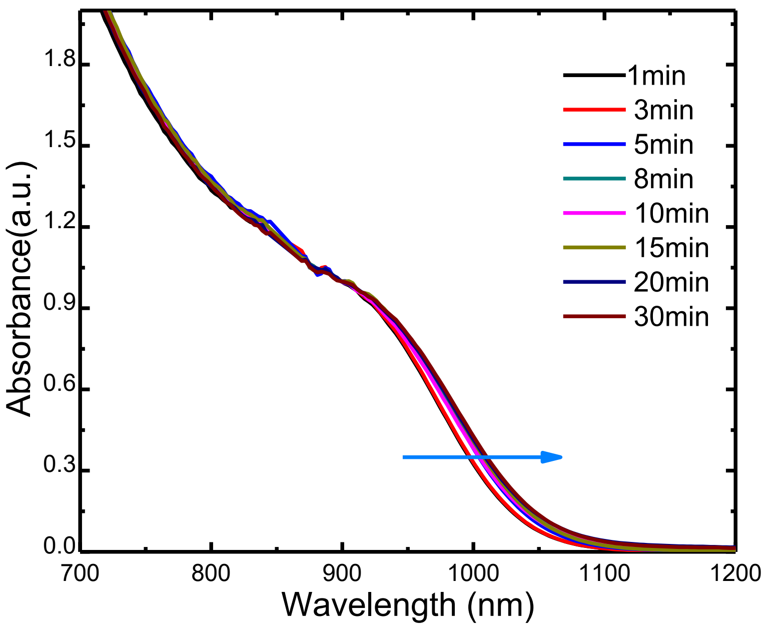

Supplementary Figure 3. Absorption of PbS-I NCs extracted from different reaction time, indicating a long size-focused time.

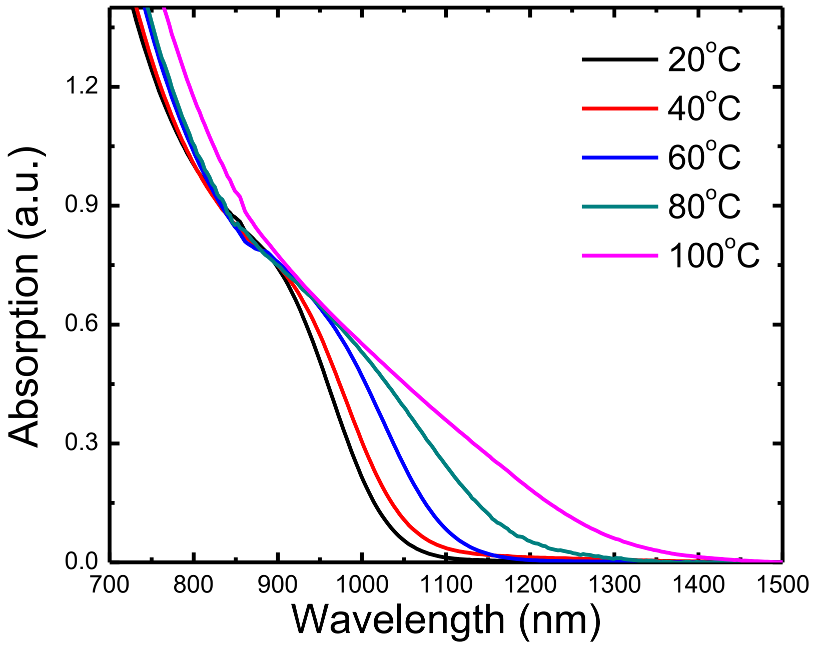

Supplementary Figure 4. Absorption of PbS-I NCs synthesized under different temperatures.

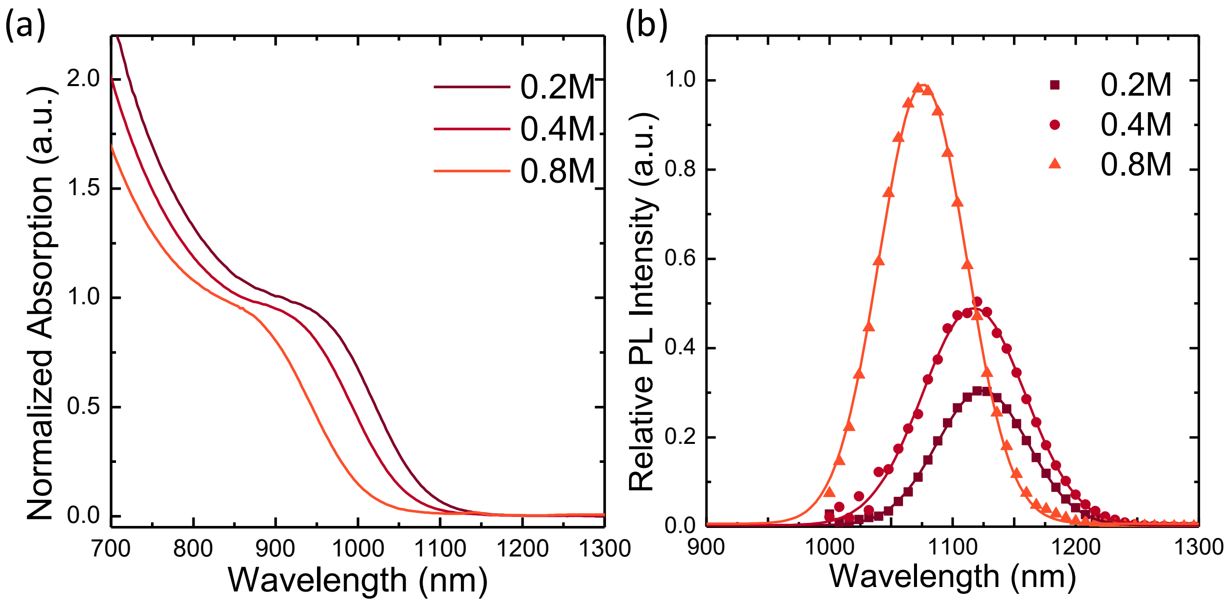

Supplementary Figure 5. Optical characterization of PbS-I NCs synthesized with different precursor concentration. Absorption (a) and photoluminescence spectrum (b) of PbS-I NCs. Pb/S precursor ratio was fixed at 4:1.

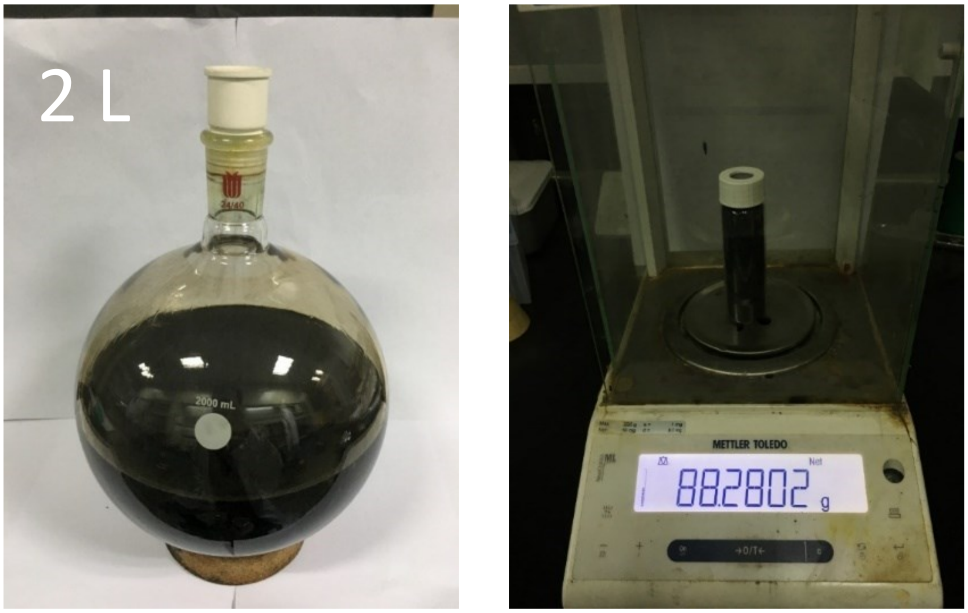

Supplementary Figure 6. Photos of scaled-up 2L synthesis. 88 g PbS-I NCs can be obtained in one-pot synthesis.

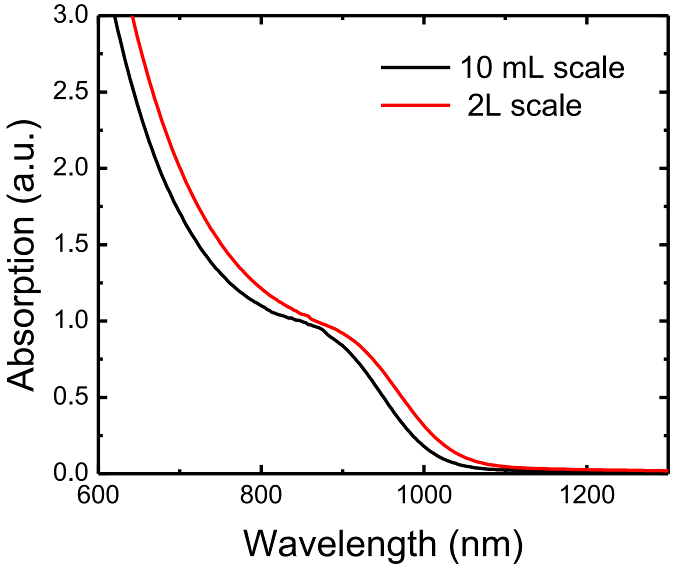

Supplementary Figure 7. Absorption spectrum of 10 mL scale synthesis and 2 L scale synthesis.

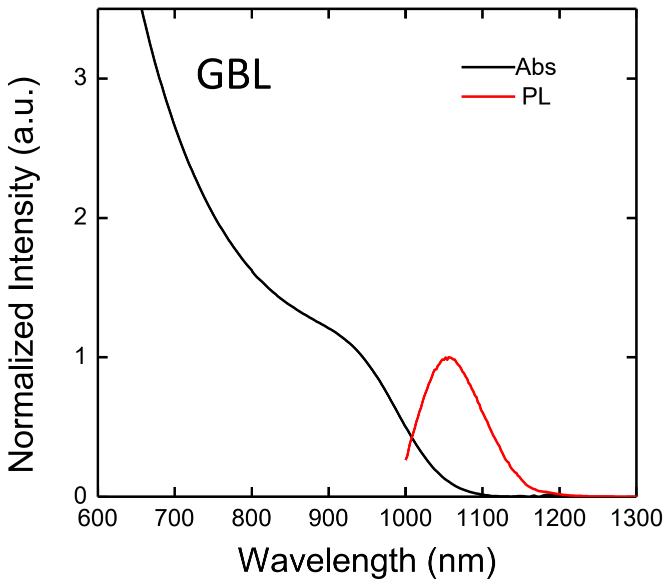

Supplementary Figure 8. Absorption and photoluminescence spectrum of PbS-I NCs synthesized with γ-butyrolactone (GBL) as the reaction solvent.

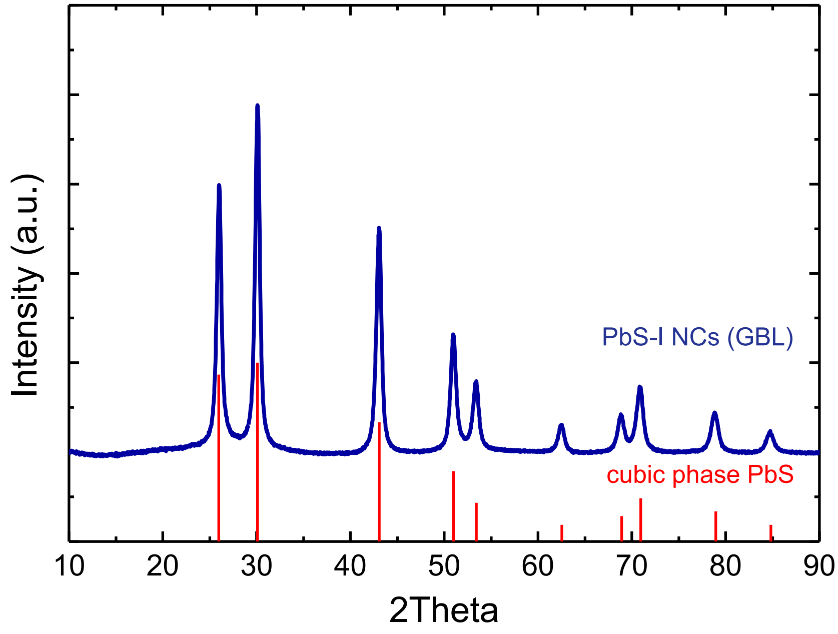

Supplementary Figure 9. X-Ray Diffraction (XRD) spectrum of PbS-I NCs synthesized with γ-butyrolactone (GBL) as the reaction solvent.

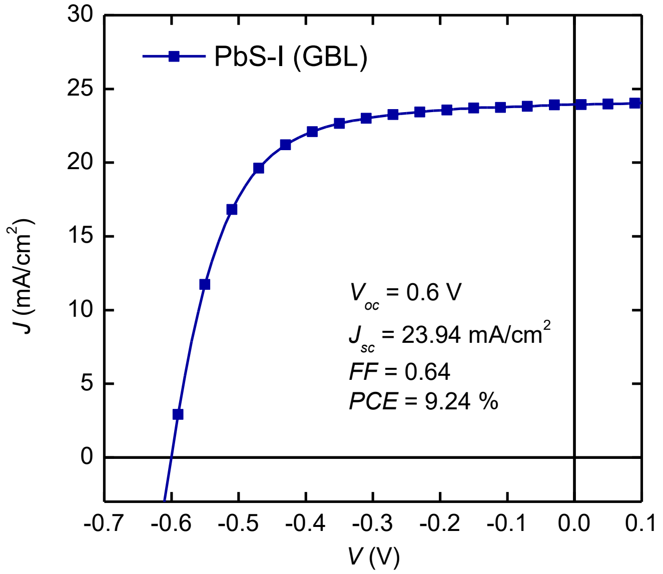

Supplementary Figure 10. *J-V* curve of solar cell based on PbS-I NCs synthesized with γ-butyrolactone (GBL) as solvent.

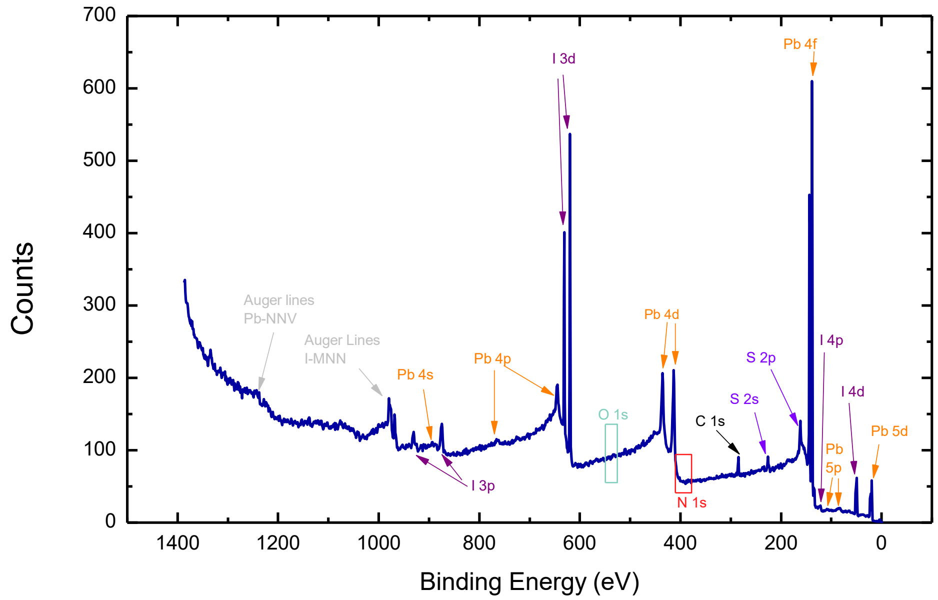

Supplementary Figure 11. X-Ray Photoelectron Spectroscopy (XPS) survey spectrum of PbS-I NCs film.

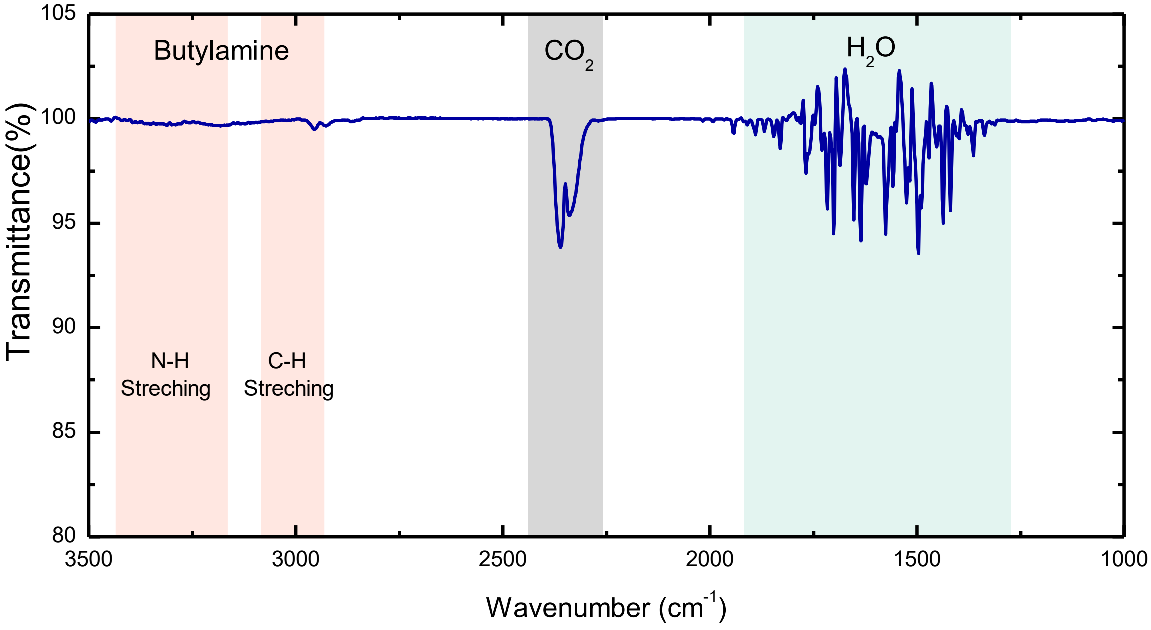

Supplementary Figure 12. Fourier-transform infrared spectrum (FTIR) of PbS-I NCs film.

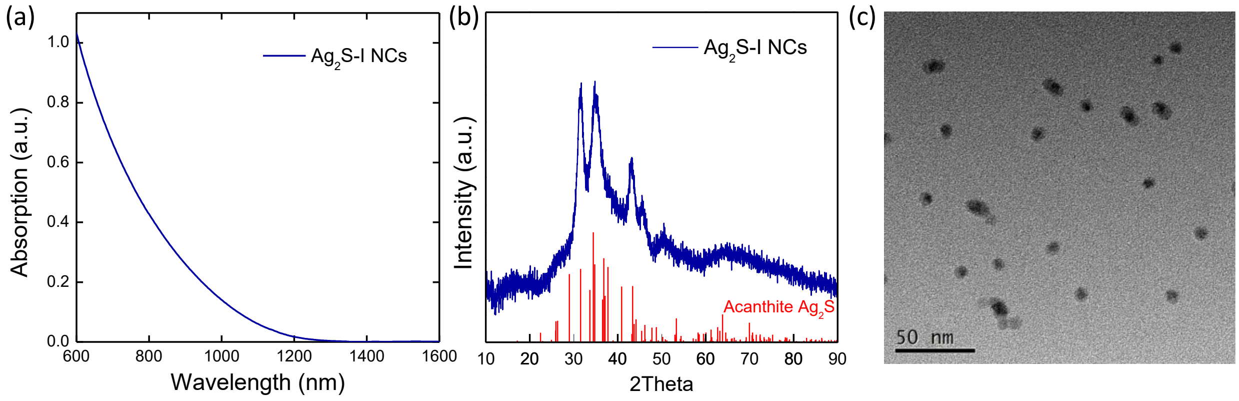

Supplementary Figure 13. Directly synthesized Ag_2S-I NCs. (a) Absorption spectrum of Ag_2S-I NCs. (b) X-Ray Diffraction (XRD) of Ag_2S-I NCs. (c) Transmission Electron Microscopy (TEM) image of Ag_2S-I NCs.

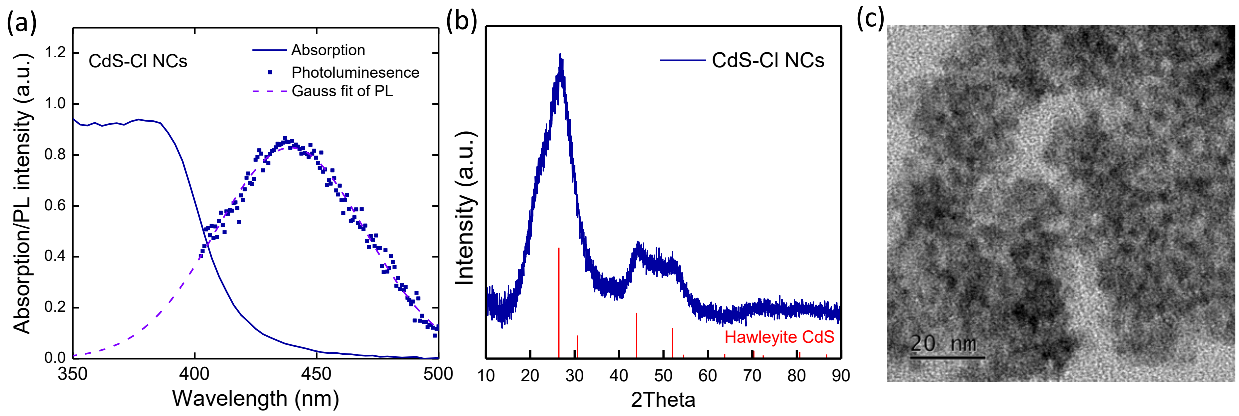

Supplementary Figure 14. Directly synthesized CdS-Cl NCs. (a) Absorption and PL spectrum of CdS-Cl NCs. (b) X-Ray Diffraction (XRD) of CdS-Cl NCs. (c) Transmission Electron Microscopy (TEM) image of CdS-Cl NCs.

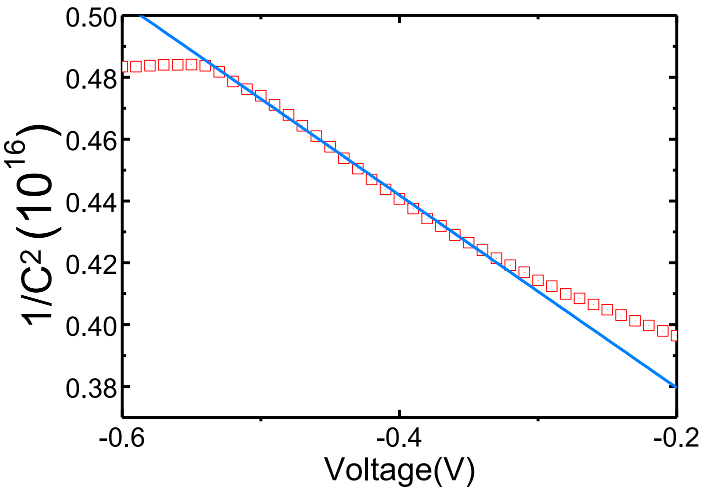

Supplementary Figure 15. Capacitance-Voltage curve of PbS-I NCs based Schottky junction. Device structure: Ag/PbS-I/Au.

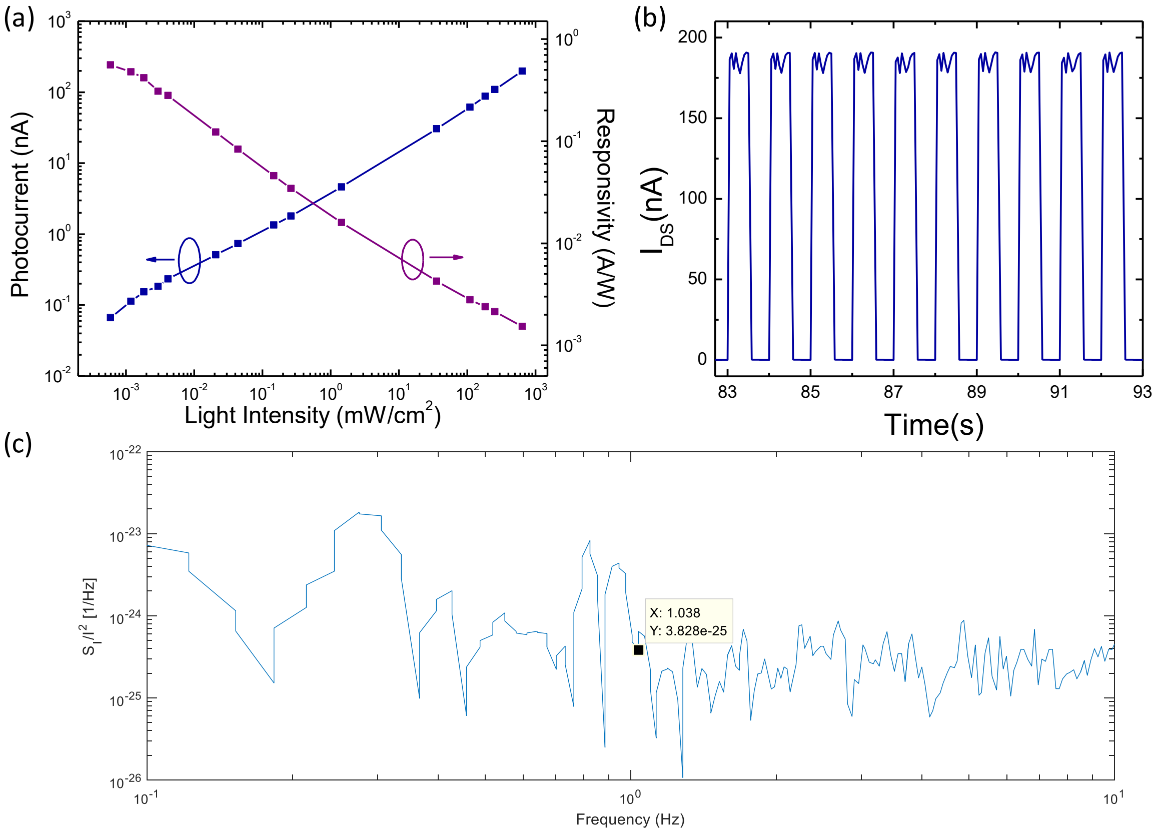

Supplementary Figure 16. Photodetector performance based on solution-phase ligand exchanged PbS NCs. (a) Photocurrent and responsivity under different light

intensity of 635 nm laser. (b) Dynamic response of the device upon on-off switching of 635 nm laser. The measurements are carried out at a drain bias of 5 V and without gate bias. (c) Noise power density of the solution-phase ligand exchanged PbS NCs photodetector.

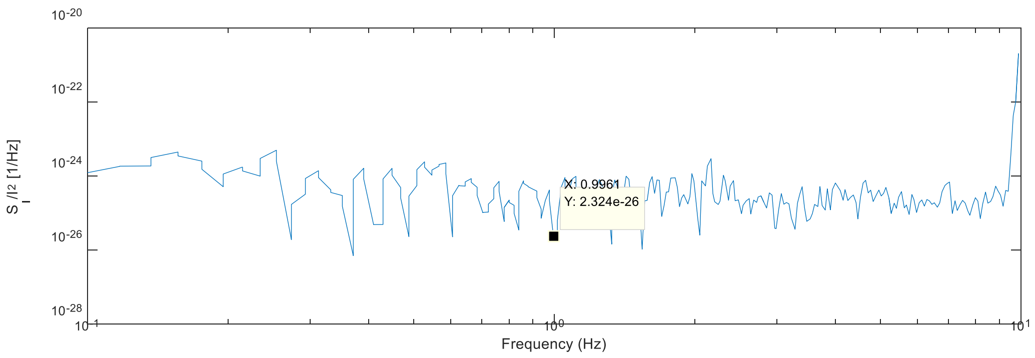

Supplementary Figure 17. Noise power density of the PbS-I NC photodetector device under the same measurement conditions.

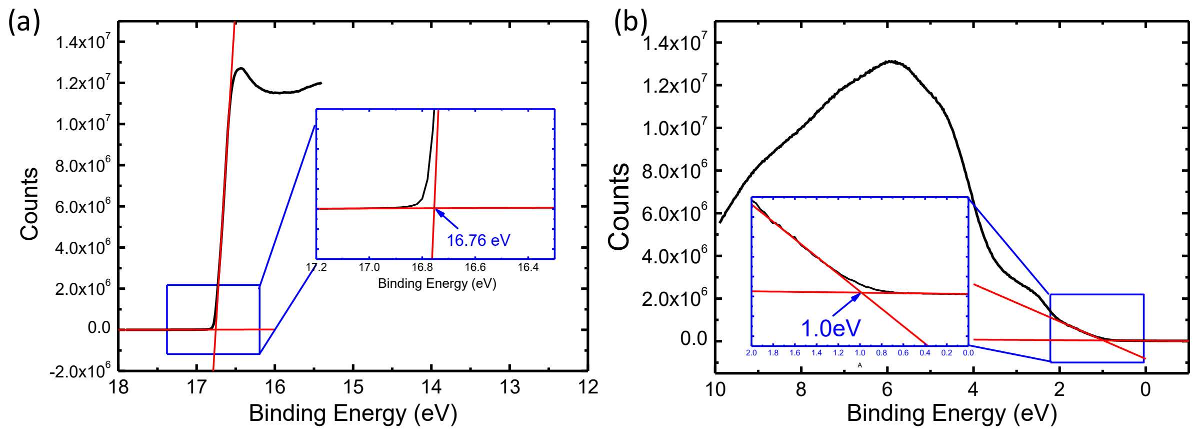

Supplementary Figure 18. Ultraviolet photoelectron spectrum (UPS) of PbS-I NCs film. The Fermi level can be calculated at 4.4 eV, the valence band maximum can be calculated at 5.4 eV.

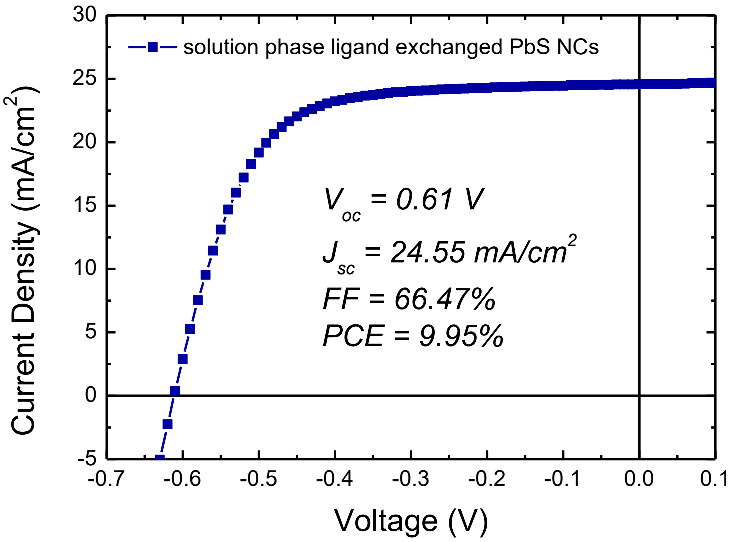

Supplementary Figure 19. *J-V* curve of solution-phase ligand exchanged PbS NCs solar cells.

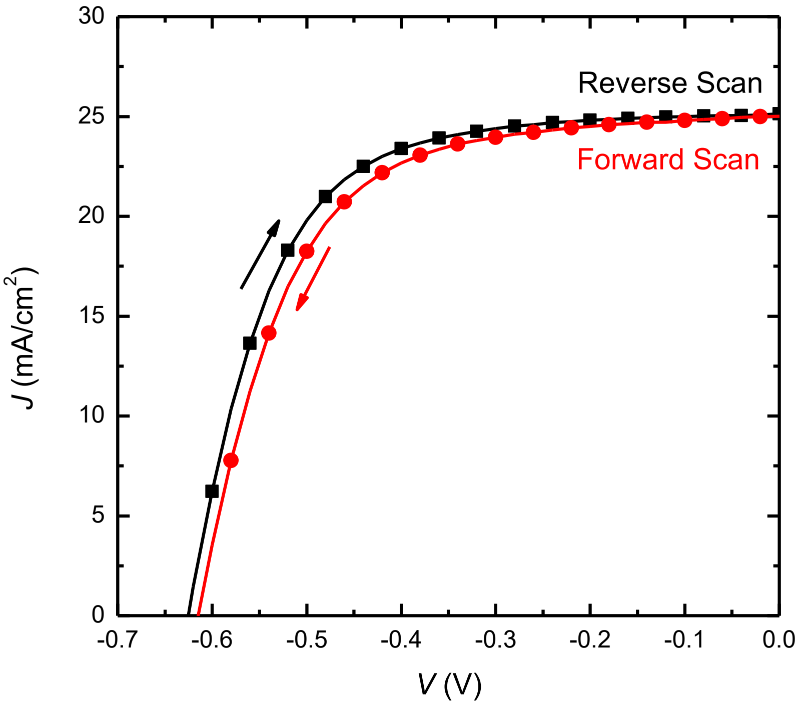

Supplementary Figure 20. *J-V* Curve of champion device (forward scan and reverse scan). Devices show slight hysteresis.

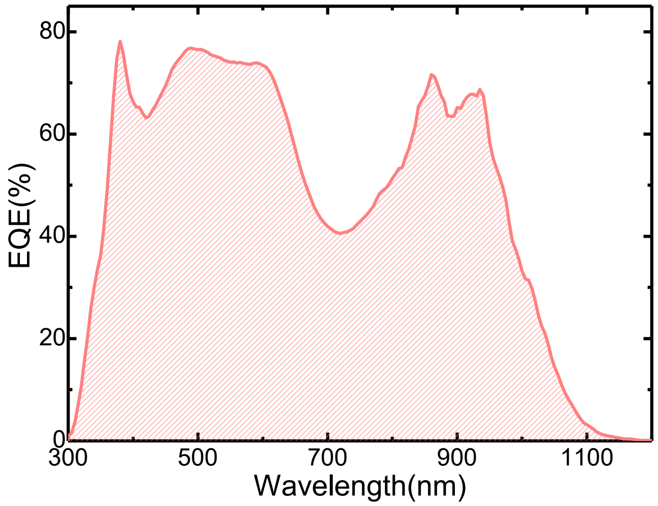

Supplementary Figure 21. External quantum efficiency (EQE) spectrum of optimized PbS-I NCs device. Integrated J_{sc} = 23.93 mA·cm^{-2}.

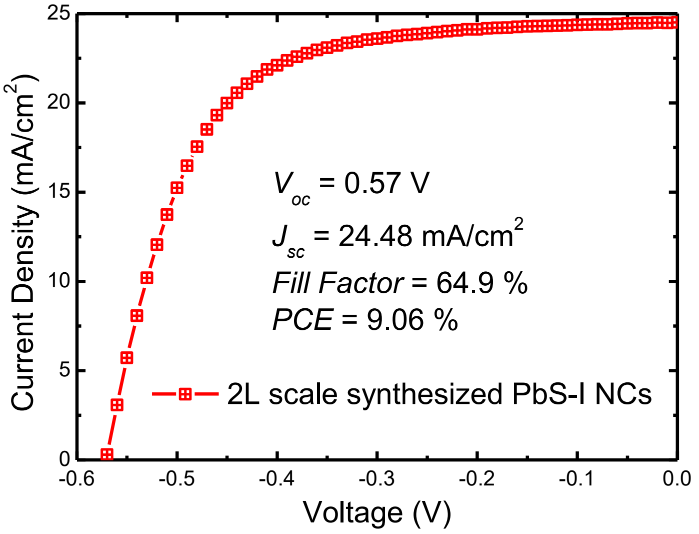

Supplementary Figure 22. *J-V* curve of solar cell based on 2L scale synthesized PbS-I NCs.

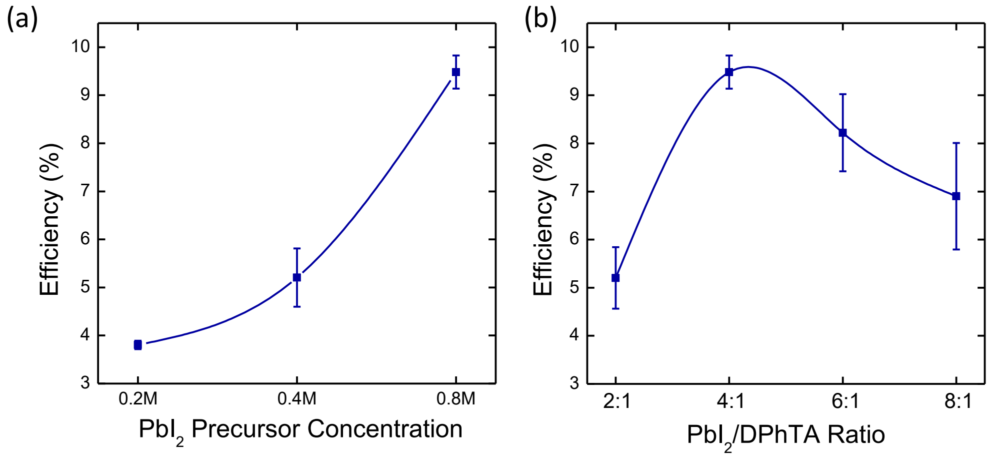

Supplementary Figure 23. Average device performance based on PbS-I NCs synthesized with different precursor concentration (a) and lead/sulphur ratio (b). The error bar in figures represent the standard deviation between different devices.

Supplementary Table 1. Chemicals needed for 88 g PbS-I NCs inks through conventional "synthesis to ligand exchange" method.

	Supplier	Purity	Price	Amount used	Cost
**$Pb(Ac)_2 \cdot 3H_2O$**	Alfa	99%	¥0.942/g	167.2 g	¥157.5
**TMS**	J&K	98%	¥160/mL	44 mL	¥7040
**OA**	Alfa	90%	¥0.385/g	308 g	¥118.5
**ODE**	Alfa	90%	¥0.343/g	2640 g	¥905.5
**Hexane**	Sinopharm Group	95%	~¥0.04/mL	60280 mL	¥2411.2
**IPA**	Sinopharm Group	95%	~¥0.04/mL	9900 mL	¥396
**Acetone**	Sinopharm Group	95%	~¥0.03/mL	5940 mL	¥178.2
**PbI_2**	Adamas	99+%	¥4.125/g	507 g	¥2091.3
**AA**	Sigma-Aldrich	>98%	¥1.54/g	34.1 g	¥52.5
**DMF**	Sigma-Aldrich	99.8% anhydrous	¥1.128/mL	11000 mL	¥12408
**Toluene**	Sinopharm Group	~95%	~¥0.04/mL	6600 ml	¥264
**Total**					¥26022.7

Supplementary Table 2. Chemicals used for 2L scale direct-synthesis of PbS-I NCs inks.

	Supplier	Purity	Price	Amount used	cost
**PbI_2**	Adamas	99+%	¥4.125/g	368.8 g	¥1521.3
**DPhTA**	Sigma-Aldrich	98%	¥8.256/g	45.6 g	¥376.5
**DMF**	Sigma-Aldrich	99.8% anhydrous	¥1.128/mL	900 mL	¥1015.2
**BA**	TCI	>99%	¥0.369/mL	100 mL	¥36.9
**Toluene**	Sinopharm Group	~95%	~¥0.04/mL	3000 mL	¥120
**Total**					¥3069.9

Supplementary Table 3. Parameters of photodetectors based on different PbS NCs.

Photoactive material	Responsivity ($A \cdot W^{-1}$)	Detectivity (*Jones*)	Noise equivalent power ($pA \cdot Hz^{-1/2}$)	Decay time (ms)
Direct-synthesized PbS-I NCs	1.5	1.4×10^{11}	0.15	< 4
Solution-phase ligand exchanged PbS NCs	0.56	1.3×10^{10}	0.62	< 4

Supplementary Note 1: Proposed reaction process:

Before discussing our direct synthesis of PbS NCs ink, we briefly review the mechanism of the typical synthesis for oleate capped PbS NCs through hot-injection reaction. For conventional hot injection synthesis method, lead oxide, lead acetate or lead halides are used as lead precursors; bis(trimethylsiliane sulphide) (TMS-S) or oleylamine (OLA)-S are used as sulphur precursor; oleic acid (OA) or OLA are used as ligands [1-4]. The reactions can be summarized as follow:

$$Pb(oleate)_2 + TMS\text{-}S \longrightarrow PbS\text{-}OA\ NCs \quad (1)$$

$$PbX_2\text{-}OLA + S\text{-}OLA \longrightarrow PbS\text{-}OLA\ NCs \xrightarrow{OA\ Exchange} PbS\text{-}OA\ NCs \quad (2)$$

In the recipe (1), the high reactive TMS-S can easily react with metal salts (e.g., halides, acetates, metal alkyls), forming metal chalcogenide NCs and TMS-X (X = halogen, acetate, alkyl) [5].

In the recipe (2), S powder is converted to thioamide, which is further converted to H_2S. The generated H_2S can react with metal precursors to form metal sulfide NCs. It has been confirmed thioacetamide dissolved in OLA can also work as sulfur precursor, which can react with Pb precursor to produce PbS NCs even at room temperature [6]. In addition, in this reaction OLA also works as surface ligand to control NCs nucleation and growth. But the OLA can only bind on NCs surface weakly, which needs to be exchanged with OA.

In our direct synthesis method,

$$PbI_2 + DPhTA \xrightarrow{BA} PbS\text{-}I\ NCs \quad (3)$$

Since PbI_2 and DPhTA cannot react in the absence of BA, it should be reasonable to propose that the ^{-}SH converted from DPhTA under alkaline condition works as the real sulfur precursor[7,8], analogous to reaction (2). Then we can propose the detailed process of our reaction as shown in Supplementary Figure 1.

In order to confirm if BA also function as ligands in our reaction system, we use TMS-S, instead of DPhTA, as the sulphur precursor. As shown in Supplementary Figure 2, PbS NCs can be obtained with TMS-S as well. This reaction can also be triggered without BA, but it can only result in black PbS precipitates with large size and serious aggregations, which indicates BA also functions as surface ligand to control the nucleation and growth in our direct ink synthesis (reaction 3).

Since BA binds on PbS NCs surface weakly and possesses low boiling point (78 ^{o}C), it will be easily removed during purification and spin-coating step. The special property of BA can ensure low residual of organic compound in the final coated film, which is beneficial for charge transport. It is also the same reason BA was used as solvent to dissolve PbS ink for solar cells fabrication[9,10].

Supplementary Note 2: Synthesis cost estimation.

Estimated cost for conventional "synthesis to ligand exchange" method:

For the hot-injection synthesis of oleic acid capped PbS NCs, 10 mmol $PbAc_2 \cdot 3H_2O$, 7 g OA, 60 g ODE, 1 mL TMSS are needed to obtain around 2.5 g PbS-OA NCs in one batch. For the purification of PbS-OA NCs, 120 mL hexane, 225 mL isopropanol (IPA) and 135 mL acetone are needed. During solution-phase ligand-exchange process, 100 mL DMF, 10 mmol PbI_2, 4 mmol ammonium acetate, 500 mL hexane, and 1 g PbS-OA NCs are needed for one batch to obtain less than 0.8 g PbS-I NCs. In order to obtain 88 g PbS-I NCs, 110 batches solution-phase ligand-exchange and 44 batches hot-injection synthesis are needed. The time for one person to do these works is estimated to be more than two weeks.

Therefore, for conventional "two-steps" method, the synthesis cost in our lab of 88 g PbS-I NC inks is ¥26022.7 based on the material cost in Supplementary Table 1, which is ¥295.7 or around $43 per gram PbS-I NCs, as shown in Supplementary Table 1. Note that the price is significant higher than the 16 $\$\cdot g^{-1}$ estimated in reference 25 because of the difference in material cost in different lab. In addition, the heating and labour cost are not taken into account yet.

Estimated cost for direct-synthesis method:

For 2 L scale synthesis, 0.8 mol PbI_2, 0.2 mol DPhTA, 900 mL DMF and 100 mL BA were used as precursors and solvents. Toluene was used as anti-solvent. As shown in Supplementary Table 2, the total cost to directly synthesize 88.28 g PbS-I NCs in our lab is only ¥3069.9, which means the price per gram PbS-I NCs is ¥34.77 or around $5.05. Based on the same cost estimation method, this price is much lower than the cost of 43 $\$\cdot g^{-1}$ for conventional PbS inks obtained in our lab. Furthermore,

the total synthesis and purification time is less than 1 hour, which means the labour cost is negligible compared to the "two-steps" method. The synthesis cost of PbS-I NCs is expected to be further largely reduced in practical application, due to the price reduction of raw materials with increasing scale.

Supplementary Note 3: Carrier density calculation.

Capacitance voltage characteristics were measured with modulation frequency and amplitude of 5 kHz and 100 mV, respectively, between -1 V to +1 V with an AC signal. The Schottky junction device of Ag/PbS-I/Au was fabricated for the calculation of dielectric constant and carrier density. The dielectric constant of PbS-I film was extracted from the following equation,

$$C = \varepsilon_0 \varepsilon_{\text{NCs}} \frac{A}{d} \tag{4}$$

Where ε_0 is the permittivity of free space, A is the area of the device and d is the thickness of PbS-I NC film. The calculated ε_{NCs} is 19.8 for PbS-I NC films.

The carrier concentration (N_{NCs}) was obtained from the Mott-Schottky analysis by fitting the linear region of $C^{-2} \sim V$ curve using following equation,

$$\frac{1}{C^2} = \frac{2(V_{\text{bi}} - V)}{A^2 \mathrm{e} \varepsilon_0 \varepsilon_{\text{NCs}} N_{\text{NCs}}} \tag{5}$$

Where e is the elementary charge, V_{bi} is the built-in potential in the device, as shown in Supplementary Figure 15. The N_{NCs} was calculated to be 8.77×10^{16} cm^{-3}.

Supplementary Note 4: Space charge limited current test for trap density.

Electron only devices were fabricated using a device structure of Ag/PbS-I/Ag. *J-V* curves of devices exhibiting space charge limited current (SCLC) in the presence of trap states show three characteristic regions. At low bias, thermally generated charges are more than the injected charge carriers and the device follows Ohm's law. At a certain voltage, the injected charges exceed the thermally generated charges and injected charges fill the trap states leading to a trap-filling SCLC behaviour. The onset voltage V_{TFL} is linearly proportional to the trap density N_{t}.

$$V_{\mathrm{TFL}} = \frac{\mathrm{e}N_{\mathrm{t}}d^2}{2\varepsilon_0\varepsilon_{\mathrm{NCs}}} \qquad (6)$$

The V_{TFL} for PbS-I NC film is 0.2 V, with a thickness of 250 nm, the trap density is calculated to be 7×10^{15} cm^{-3}.

Supplementary References

1. Hines, M.A. & Scholes, G.D. Colloidal PbS nanocrystals with size-tunable near-infrared emission: Observation of post-synthesis self-narrowing of the particle size distribution. *Adv. Mater.***15**, 1844-1849 (2003).
2. Zhang, J., Gao, J., Miller, E.M., Luther, J.M. & Beard, M.C. Diffusion-controlled synthesis of PbS and PbSe quantum dots with in situ halide passivation for quantum dot solar cells. *ACS Nano* **8**, 614-622 (2014).
3. Weidman, M.C., Beck, M.E., Hoffman, R.S., Prins, F. & Tisdale, W.A. Monodisperse, air-stable PbS nanocrystals via precursor stoichiometry control. *ACS Nano* **8**, 6363-6371 (2014).
4. Chuang, C.H., Brown, P.R., Bulovic, V. & Bawendi, M.G. Improved performance and stability in quantum dot solar cells through band alignment engineering. *Nat. Mater.* **13**, 796-801 (2014).
5. MacDonald, D.G. and Corrigan, J.F., Metal chalcogenide nanoclusters with 'tailored' surfaces via 'designer' silylated chalcogen reagents. *Phil. Trans. R. Soc. A,* **368**, 1455-1472 (2010).
6. Thomson, J.W., Nagashima, K., Macdonald, P.M. & Ozin, G.A. From sulfur−amine solutions to metal sulfide nanocrystals: peering into the oleylamine−sulfur black box. *J. Am. Chem. Soc.*, **133**, 5036-5041 (2011).
7. García-Rodríguez, R., Hendricks, M.P., Cossairt, B.M., Liu, H. & Owen, J.S. Conversion reactions of cadmium chalcogenide nanocrystal precursors. *Chem. Mater.* **25**, 1233-1249 (2013).
8. Walter, J. L., et al. A study of thiourea and substituted thiourea analogs by the Bjerrum titration method. *J. Am. Chem. Soc.* **78**, 5560-5562 (1956).
9. Liu, M. et al. Hybrid organic-inorganic inks flatten the energy landscape in colloidal quantum dot solids. *Nat. Mater.* **16**, 258-263 (2017).
10. Xu, J. et al. 2D matrix engineering for homogeneous quantum dot coupling in photovoltaic solids. *Nat. Nanotechnol*. **13**, 456-462 (2018).
