# Supplementary material for: Room-temperature direct synthesis of semi-conductive PbS nanocrystal inks for optoelectronic applications
Source: Nat Commun. 2019 Nov 13;10:5136. doi: 10.1038/s41467-019-13158-6 (PMC6853884; doi:10.1038/s41467-019-13158-6)
Supplement: Supplementary file 3 — Reporting Summary [file 41467_2019_13158_MOESM3_ESM.pdf]

nature research

Corresponding author(s): Wanli Ma

Solar Cells Reporting Summary

Nature Research wishes to improve the reproducibility of the work that we publish. This form is intended for publication with all accepted papers reporting the characterization of photovoltaic devices and provides structure for consistency and transparency in reporting. Some list items might not apply to an individual manuscript, but all fields must be completed for clarity.

For further information on Nature Research policies, including our data availability policy, see Authors & Referees.

Experimental design

Please check: are the following details reported in the manuscript?

1. Dimensions

Area of the tested solar cells	☒ Yes ☐ No	0.0725 cm2, stated in Methods section
Method used to determine the device area	☒ Yes ☐ No	The overlap of ITO and gold electrodes.

2. Current-voltage characterization

Current density-voltage (J-V) plots in both forward and backward direction	☒ Yes ☐ No	Supplementary Figure 23 on page 14 of the Supplementary information
Voltage scan conditions<br>*For instance: scan direction, speed, dwell times*	☒ Yes ☐ No	Voltage scan conditions are described in “Methods” section of the main text.
Test environment<br>*For instance: characterization temperature, in air or in glove box*	☒ Yes ☐ No	Test environment is described in “Methods” section of the main text.
Protocol for preconditioning of the device before its characterization	☐ Yes ☒ No	No preconditioning protocol like encapsulation was used before characterization.
Stability of the J-V characteristic<br>*Verified with time evolution of the maximum power point or with the photocurrent at maximum power point; see ref. 7 for details.*	☐ Yes ☒ No	Not applicable.

3. Hysteresis or any other unusual behaviour

Description of the unusual behaviour observed during the characterization	☒ Yes ☐ No	Slight hysteresis is described in Supplementary information.No other unusual behaviors were observed in the testing.
Related experimental data	☒ Yes ☐ No	Supplementary Figure 23 on page 14 of the Supplementary information

4. Efficiency

External quantum efficiency (EQE) or incident photons to current efficiency (IPCE)	☒ Yes ☐ No	Supplementary Figure 20 on page 12 of the Supplementary information
A comparison between the integrated response under the standard reference spectrum and the response measure under the simulator	☒ Yes ☐ No	Page 10 of the main text
For tandem solar cells, the bias illumination and bias voltage used for each subcell	☐ Yes ☒ No	N.A.

5. Calibration

Light source and reference cell or sensor used for the characterization	☒ Yes ☐ No	It is described in “Methods” section of the main text.
Confirmation that the reference cell was calibrated and certified	☒ Yes ☐ No	It is described in “Methods” section of the main text.

Calculation of spectral mismatch between the reference cell and the devices under test

- [ ] Yes
- [x] No

No mismatch calculation was performed.

6. Mask/aperture

Size of the mask/aperture used during testing

- [ ] Yes
- [x] No

Mask/aperture is not used.

Variation of the measured short-circuit current density with the mask/aperture area

- [ ] Yes
- [x] No

Mask/aperture is not used.

7. Performance certification

Identity of the independent certification laboratory that confirmed the photovoltaic performance

- [ ] Yes
- [x] No

A certified efficiency is not relevant for the scope of this work.

A copy of any certificate(s)
Provide in Supplementary Information

- [ ] Yes
- [x] No

No certificate. Please see above.

8. Statistics

Number of solar cells tested

- [x] Yes
- [ ] No

Page 9-10 and Figure 5c in main text.

Statistical analysis of the device performance

- [x] Yes
- [ ] No

Page 9-10 and Figure 5c in main text.

9. Long-term stability analysis

Type of analysis, bias conditions and environmental conditions
For instance: illumination type, temperature, atmosphere humidity, encapsulation method, preconditioning temperature

- [x] Yes
- [ ] No

Page 10 and Figure 5d in main text.
